# Quality attributes of *fufu* in South‐East Nigeria: guide for cassava breeders

**DOI:** 10.1111/ijfs.14875

**Published:** 2020-12-16

**Authors:** Ugo Chijioke, Tessy Madu, Benjamin Okoye, Amaka Promise Ogunka, Mercy Ejechi, Miriam Ofoeze, Chukwudi Ogbete, Damian Njoku, Justin Ewuziem, Confidence Kalu, Nnaemeka Onyemauwa, Blessing Ukeje, Oluchi Achonwa, Lora Forsythe, Geneviève Fliedel, Chiedozie Egesi

**Affiliations:** ^1^ National Root Crops Research Institute (NRCRI) Umudike, Umuahia Nigeria; ^2^ Natural Resources Institute University of Greenwich Central Avenue Chatham Maritime Kent ME4 4TB UK; ^3^ CIRAD UMR QUALISUD Montpellier F‐34398 France; ^4^ Qualisud, Univ Montpellier, CIRAD, Montpellier SupAgro, Univd'Avignon Univ de La Réunion Montpellier 34398 France; ^5^ International Institute of Tropical Agriculture (IITA) Oyo Road Ibadan Nigeria; ^6^ Department of Global Development Cornell University Ithaca NY 14853 USA

**Keywords:** Breeding, consumers, *fufu*, gender, preferred traits, processing, quality characteristics, varieties

## Abstract

*Fufu* is a popular traditional fermented wet paste food product from cassava. We examined consumer preferences and quality attributes of *fufu* in Abia and Imo States of South‐East Nigeria, with special attention to gender differences, for the purpose of providing guidance to breeders. Data were analysed by the use of descriptive and inferential statistics. Participants for the interview were randomly selected from a list of farmers in the study area. Individual (II) interviews were conducted among eighty participants comprising twenty‐six men (32.5%) and fifty‐four women (67.5%). Preferences along the food chain from raw roots to final product were also obtained. Major traits influencing gender‐specific consumer preferences are related to appearance, texture and smell. Smoothness, not sticky, easy to swallow and drawability of *fufu* appear to be major traits that drive acceptance by both men and women. Big roots and smooth skin are prioritised for raw material. Some quality characteristics are conditioned largely by variety traits, while others can be modified by adjusting the processing methods. The complexity of producing high‐quality *fufu* makes it imperative to introduce a multidisciplinary approach into breeding programmes.

## Graphical abstract

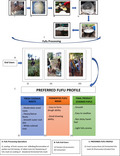

## Introduction

Nigeria is the world’s largest producer of cassava, hosting a diverse array of cassava farmers and processors, with the large majority being small‐scale operators (Forsythe *et* *al*., 2016). Major cassava‐based food products consumed in Nigeria include the following: *gari*, *fufu* and *lafun*. Most of these products are made and consumed locally by farming households themselves (IITA, 2012). Fufu is a traditional Nigerian fermented food product in southern, western and eastern Nigeria and some other parts of West Africa (Rosales‐soto *et* *al*., 2016). It is usually described as a ‘wet paste food product’ and ranks second after *gari* as a food product from cassava (http://www.cassavabiz.org/postharvest/fufu). In some parts of Nigeria, it is also called *utaraakpu* (Owolarafe *et* *al*., 2018). Uche (2016) reported that *fufu* has higher profit, gross margin, mark‐up and a better monetary prospect. According to Sanni *et* *al*. (1998), late in the 20^th^ century, 60% of all the cassava harvested across Nigeria was used in processing *fufu*, and only 5% for *gari*. However, the preference and consumption pattern has been reversed between *fufu* and *gari* in recent times, because of *fufu*’s poor shelf life and tedious processing methods. *Fufu* is ranked next to *gari* as an indigenous food in southern Nigeria (Egwim *et* *al*., 2013), and it is popular in many parts of West Africa (Uyoh *et* *al*., 2009). *Fufu* flour as a convenience food and staple is increasingly becoming very popular in West Africa (Johnson *et* *al*., 2006).

*Fufu* is usually processed by households and rural processors whose practices may differ by culture and region. Fermentation is a key component of *fufu* production, an important step to detoxify the cassava pulp (i.e. degrade cyanogenic glucosides), develop the characteristic aroma and flavour of the *fufu*, and also help in preserving it (Flibert *et* *al*., 2016). *Fufu* is produced by first, peeling and washing the cassava roots, and cutting them into smaller chunks. The method of soaking/steeping of roots differ among states and processors in South‐East Nigeria. Soaking, steeping or fermentation of the cut roots may be carried out either by continuous soaking of chunked roots for a period of 3–5 days of fermentation (Mokemiabeka *et* *al*., 2011), or by washing and grating of the soaked root after 48hrs of fermentation, followed by re‐steeping of grated roots (Omodamiro *et* *al*., 2012). The fermented roots or mash are finally sieved, and dewatered to obtain the wet paste. The sour taste, flavour, appearance and texture are mainly recognised as determinants of *fufu* acceptance and quality (Bamidele *et* *al*., 2015). The variations in processing methods and differences in physico‐chemical properties of cassava varieties alter the texture and organoleptic properties of the cooked *fufu* (Akingbala *et* *al*., 1991). Furthermore, Bechoff *et* *al*. (2018) and Asrat *et* *al*. (2010) noted that gender‐specific crop trait preferences are rarely considered or prioritised in most breeding programs. These complexities involved in the processing of the product (*fufu*) make it imperative for the need to introduce a multidisciplinary approach for breeding varieties that meet end user needs for *fufu*. The study therefore described the quality characteristics that drive purchase and utilisation of fresh cassava roots for *fufu* processing, preferred quality characteristics of cassava root for processing of fermented wet *fufu* mash, cooking properties of the intermediate *fufu* product and quality characteristics of *fufu* during consumption.

## Materials and methods

### Study area

**Figure 1:**
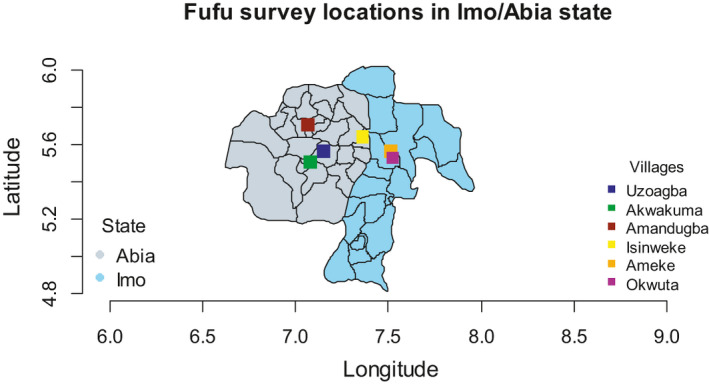
Survey locations in Imo and Abia States of Nigeria.

The study was conducted in Imo and Abia States in South‐East region of Nigeria, selected based on intensity of cassava production (Fig. 1). Imo and Abia States are made up of three Agricultural Zones each. Imo State is divided into twenty‐seven administrative units called local government areas (LGAs), which are grouped into three Agricultural Zones of Owerri, Okigwe and Orlu, and seventeen LGAs for Abia State (grouped into three Agricultural Zones of Aba, Ohafia and Umuahia). The climate can generally be described as tropical – in the humid rainforest agro‐ecological zone – with clearly defined wet and dry seasons. Smallholder crop and livestock farming is the predominant occupation of the people (Anderson *et* *al*., 2017). Generally, a smallholder farmer is involved in cultivating a small piece of land, cultivating food crops, sometimes with small varieties of cash crops (Thorpe & Muriuki, 2001; Herrero *et* *al*., 2014). In many localities, smallholder farmers practise mixed crop‐livestock farming, whereby the number of large ruminants kept is around 3–5 (Thorpe & Muriuki, 2001). The major crops produced include the following: yam, cassava, rice, maize, cocoyam, cowpea and tomatoes. *Fufu* is a principal food staple in Imo and Abia States.

### Sampling procedure

A multistage sampling procedure was used to select sample respondents to identify traits for a high‐quality crop (cassava) and product (*fufu*), among the cassava‐producing and cassava‐consuming households in the region (Forsythe *et* *al*., 2020). In the first stage, three Agricultural Zones in Imo State and one in Abia State were selected for the study based on level of fufu processing and consumption. In the second stage, four communities in Imo and two in Abia States were selected, also based on intensity of fufu processing and convenience. In the last stage, participants for the interview were randomly selected from a list of farmers provided by the Agricultural Development Programme (ADP) in the communities. ADPs aim at increasing food production for rural dwellers, and raising the income level of small‐scale farmers, by making provision for improved seeds, fertiliser, pesticides, credit facilities and infra‐structural facilities (Ajayi & Ajala, 1999). Individual interviews (II) were conducted, comprising ten participants in each community of Imo State (giving forty participants in four communities) and twenty in each community (forty participants) of Abia State, giving a total of eighty participants (fifty‐four female and twenty‐six male).

### Data collection and analysis

The data for the study were collected from primary sources by the use of a well‐structured questionnaire. The questionnaires were designed to collect a range of information, including household structure, crop production, sales, utilisation and consumption of cassava with special emphasis on *fufu*. The Pivot Table function available in Microsoft Excel was used for analysis. The Pivot Table feature is a user‐friendly and easy‐to‐use tool, which is relevant for the kinds of analysis, which is required in a trait preference evaluation, following FAO (2016). Characteristics of preferred traits were assigned weights according to how they were prioritised and the importance they were given by respondents following Forsythe *et* *al*. (2020). To aggregate the characteristics into one table, we gave each of them weights according to how they were prioritised, and the importance they were given by respondents. This is to identify what characteristics to be prioritised. Comparison of different priorities for men and women was also depicted to see how the important characteristics differed. To apply weights, frequency (count) for the most important characteristic (1^st^ priority) was multiplied by 3, the frequencies for the second priority characteristic by 2 and the frequencies for the third priority characteristic by 3. The results are weighted scores summarised and ranked as:

(1) $$Y=\sum_{k=i}^{n}\left(N1\times 3\right)+\left(N2\times 2\right)+\left(N3\times 1\right)$$

where *Y* = summation of weighted scores, *N*1 = total number of respondents indicating highest (3) preferred traits, *N*2 = total number of respondents indicating second (2) most preferred traits, *N*3 = total number of respondents indicating third (1) most preferred traits, For each characteristic, total ‘points’ from men and women were summed up to get a final score and characteristics sort by descending final scores, and ranked. Results were then presented in tables, graphs and flow diagrams from which inferences were drawn.

## Results and discussion

### Preferred and less‐preferred root quality traits that drive selection of cassava varieties for fufu processing

**Figure 2:**
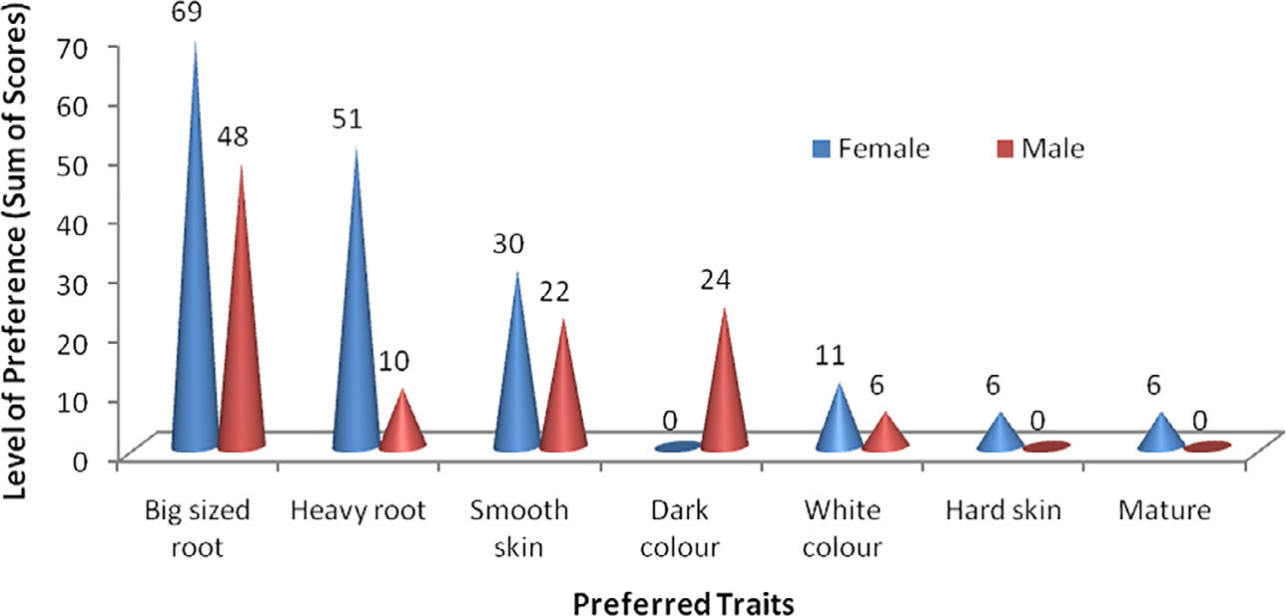
Preferred characteristics of good cassava roots for fufu when buying in the market.

**Figure 3:**
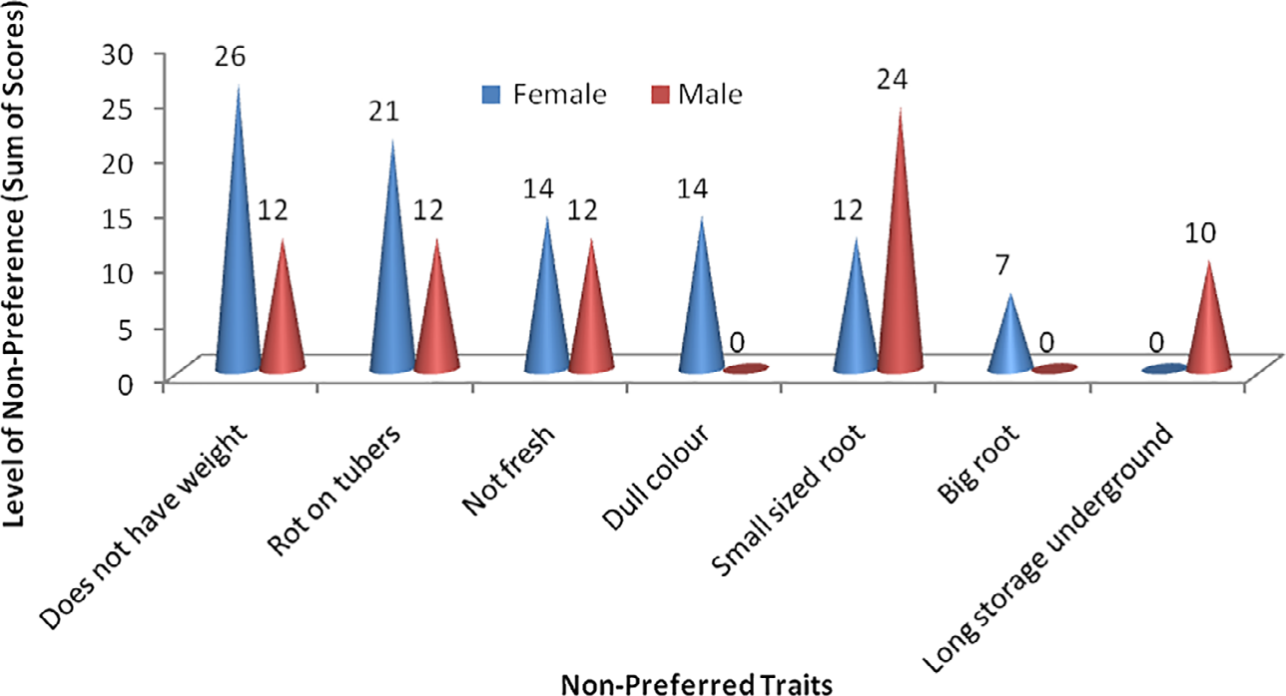
Characteristics of cassava roots in the market expected to give a less‐preferred fufu product.

The evaluation of the preferred and less‐preferred quality characteristics that drive purchase and utilisation of fresh cassava roots for *fufu* processing within South‐East Nigeria is shown in Figs 2 and 3. The results show divergent views in choice of trait preferences among the male and female participants. Four root quality attributes were identified as the main traits of preference, and they include the following: root size (moderately sized roots equivalent to 1 L capacity bottled water in diameter), heaviness of the root (weight/density of root when held by the hand), appearance/smoothness of root skin (dark coloured peel and roots without rough/wrinkle skin) and colour of root flesh (white coloured root flesh; without dark discoloration). In contrast, bad root colour (dark/multiple‐coloured striped roots), light foamy weight roots (bread‐like in texture), small‐sized roots, fibrous roots and roots with high moisture content were identified as less‐preferred traits that result in cooked *fufu* of low quality.

Size of root appears to be an important raw material (cassava root) trait for both male and female respondents. It was observed that although male and female respondent’s assigned high weight (sum of scores of 48 and 69, respectively) to root size, reasons for this preference differs by gender (Fig. 2). According to the female respondents, the preferred moderate‐size roots were easy to peel, saved operation time and mitigated drudgery (Table S1). This is in agreement with the study by Egbeocha *et* *al*. (2016) and Jimoh *et* *al*. (2016), who stated that size of tubers is one of the factors that is responsible for the demanding nature of peeling as a unit operation during processing. Furthermore, Jimoh and Olukunle (2012) reported that peeling efficiency was higher for larger‐sized cassava roots compared with smaller ones. According to the respondents, small‐sized roots are associated with inefficient peeling operations, resulting in peel loss and dark coloured intermediate and final product (cooked fufu). This study therefore reveals that selection of root size as the most important root quality attribute for the female cassava root buyers in Abia and Imo States is based on its influence on processing operation such as peeling and colour of derived products (intermediate and cooked *fufu*). On the contrary, the choice of root size by the male respondents seemed to be driven by direct economic gain obtained by buying the fresh roots, an activity where men are the major key actors (Bentley *et* *al*., 2017). According to González & Johnson (2009), size of cassava is a key determinant of its market price. The author reported that the bigger the root size, the higher the market price. Interestingly, producing cassava with preferred root traits do not imply an increase in production cost (González & Johnson, 2009). Our findings are in agreement with the study of Teeken *et* *al*. (2018), who indicated that gender roles are a strong determinant of preference for traits in cassava.

Heaviness is another trait driving the adoption of cassava varieties for *fufu* processing in South‐East Nigeria, and may be related to root weight and density. Gender differences were also observed in the sum scores for this trait, females (51) showed higher interest in the trait compared with their male (10) counterparts. The respondents described the term ‘heaviness’ with morphological features as seen in yam varieties that have stout and sturdy shape (see Table S1). The results show that the preferred trait ‘heavy root’ can be compared with a less‐preferred trait ‘roots that do not have weight’, referring to lightweight root, which was described by respondents as cassava roots with bread‐like texture. These traits [heaviness or lightweight of the cassava root (density)] were linked to the colour of the intermediate fufu product (fermented *fufu* mash). According to the respondents, lightweight roots float in the fermenting media during retting and develop dark brown colour, which subsequently affects the colour of the intermediate and final *fufu* product (Table S1).

Appearance/smoothness of root skin is another preferred root quality trait driving selection of fresh cassava roots for *fufu* processing. In describing appearance of the root, the respondents stated that roots with dark coloured peel and regular shape are preferred. According to the respondents, dark coloured peel is an indication that the cassava root is matured. Maturity of the cassava root could be linked to yield of *fufu*. The study by Baafi & Safo‐Kantanka (2007) reported an increase in starch yield of cassava varieties of age 12–13 months. Smoothness of the root skin on the other hand is characterised by the absence of wrinkles and roughness. According to the respondents, smoothness of the root skin attracts buyers and encourages ease of peeling. Egbeocha *et* *al*. (2016) and Jimoh *et* *al*. (2016) in their study reported that irregularity in the appearance of tubers is one of the factors responsible for the absence of efficient cassava peelers in Nigeria. This study therefore reveals a link between appearance and smoothness of cassava root to yield of end product and ease of peeling.

White colour of the inner cassava flesh is another trait that was highly rated as seen in the result (Figs 2 and 3). According to the respondents, over 90% of consumers prefer white coloured *fufu*. Ayetigbo *et* *al*. (2018) reported that colour of cassava flesh is retained in the derived products. Hence Bechoff *et* *al*. (2018) reported that yellow cassava flesh arising from the presence of carotenoids in the root results in yellow coloured intermediate and cooked *fufu*. By implication, the use of non‐white coloured cassava flesh for *fufu* processing will result in intermediate and cooked *fufu* with non‐white colour. This will invariably reduce the acceptance of such *fufu* within the study area. However Sanni *et* *al*. (1998) reported that *fufu* of good quality will either have a creamy‐white, grey or yellow colour. Tomlins *et* *al*. (2007) further reported that *fufu* flour should be creamier in appearance to increase their acceptability. Flesh colour has therefore become vital in the selection of cassava for food (Vimala *et* *al*., 2010).

### Preferred characteristics of cassava root for processing of fermented wet fufu mash

**Figure 4:**
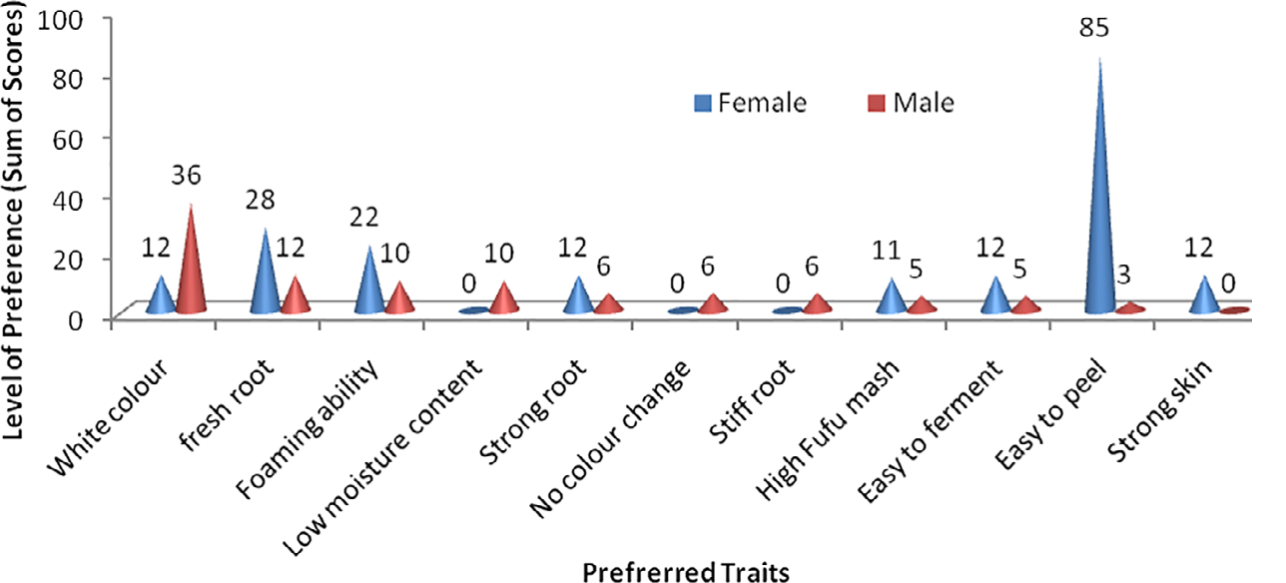
Preferred characteristics of cassava roots to be processed into fufu.

The results in Fig. 4 show preferred quality characteristics of cassava root for processing of fermented wet *fufu* mash. The results revealed gender dissimilarities in scoring of the preferred processing traits. The most outstanding preferred traits for the females were as follows: ‘easy to peel’ (85), ‘freshness of roots’ (28) (indicating absence of root rot and wound) and ‘root foaming or retting ability’ (22). The traits of interest for the males were ‘white colour’ (36) and ‘freshness of roots’ (12). Generally, results from the survey showed that the female respondents attribute more to issues related to processing ability of cassava roots for *fufu* processing compared to their male counterparts. These findings indicate that women are the major processors of fermented *fufu* mash within the study area (Teeken *et* *al*., 2018). The traits that were highly preferred by the females are directly linked to certain *fufu* processing operations such as peeling and fermentation of peeled roots. Indigenous knowledge and experience acquired by female processors over years on the effect of these traits on the wet mash, and end product (cooked *fufu*) guide selection of these traits.

According to the female respondents, the use of cassava varieties that are ‘easy to peel’ maximises the efficiency of labour and time needed to carry out other unit operations such as washing and grating. Barati *et* *al*. (2020) reported that increase in peeling efficiency is associated with increased peeled surface area and reduced peel loss. This enables the white colour of the flesh to dominate over the dark colour of the peel. Previous studies indicate that cassava peels contain certain phytochemicals such as phenols and tannins responsible for discoloration of intermediate and final cassava products such as cassava flour and *fufu* (Hongbete *et* *al*., 2009; Bindzi *et* *al*., 2014). Mokemiabeka *et* *al*. (2011) also reported that *fufu* from well‐peeled cassava had brighter white colour compared with *fufu* from unpeeled roots. This was attributed to lower tannin content in the peeled roots. Colour, according to Awoyale *et* *al*. (2018), is a major trait that drives visual appeal and acceptance of cassava products by consumers. This suggests that selection of ‘easy‐to‐peel’ cassava varieties by end users may be due to increased efficiency during manual peeling of such varieties, which invariably reduces tannin and phenol content, resulting in the production of intermediate and final *fufu* with desirable colour attribute.

Freshness of the root indicated by absence of rots/wounds on the roots is another important cassava root quality trait (Fig. 4). This was associated with the presence of ‘milkish’ white sap at the proximal end of the root. This processors’ preferred trait was also linked to the preferred traits (ease of peeling and high retting ability) by the respondents. The preference for freshly harvested roots compared with stored ones may be linked to the onset of post‐harvest physiological deterioration (PPD) of roots shortly after harvest (Zainuddin *et* *al*., 2018). PPD is associated with certain undesirable features such as vascular streaking, discoloration of roots, reduction in starch quality and shelf life, increased water loss and sugar content (Opara, 1999; Buschmann *et* *al*., 2000; Sánchez *et* *al*., 2006; Opara, 2009; Zainuddin *et* *al*., 2018). Furthermore, Swain (1979) and Rickard (1986) revealed that adverse effect of storing cassava roots could be linked to the synthesis of antinutritional compounds such as polyphenols and tannins, which eventually results in discoloration of the intermediate and cooked *fufu*. Additionally, Ampe *et* *al*. (1994) reported that storage of roots prior to retting slightly increased the production of lactate and ethanol during retting, resulting in decreased acceptability of *fufu*. From these previous studies, it could be deduced that the preferred trait ‘fresh root’ correlates positively with yield of intermediate and final product and quality in terms of colour and acceptability of *fufu*. Hence, Ampe *et* *al*. (1994) recommended that retting should be performed with freshly harvested and peeled roots. The study by Omosuli *et* *al*. (2017) also stated that storage of cassava roots leads to increase in peel loss, decrease in yield of *fufu* flour and cyanogenic potential. According to the respondents, freshness of root is also associated with appreciable moisture content, which is an important factor that facilitates peeling and encourages growth of microorganisms essential for rapid retting of the roots. This implies that the preferred trait ‘freshness of root’ is intertwined with other preferred processing traits ‘ease of peeling’ and ‘high retting ability’, and hence its importance of this root quality trait to processors and other *fufu* end users.

Foaming or retting ability (easy to ferment) of soaked roots is another preferred processing trait of cassava root mentioned by the female respondents (22) (Fig. 4). It is characterised by the presence of multiple bubbles covering the soaked roots in the fermenting vessel, and it is a key step in *fufu* processing (Sanni *et* *al*., 1998). Variability in *fufu* quality has been attributed to various local practices during retting stage of processing (Sanni *et* *al*., 1998). According to Obilie *et* *al*. (2004) and Ampe *et* *al*. (1994), retting is used to reduce cyanogenic compounds, and to improve the organoleptic quality of cassava by‐products such as *fufu*. Additionally, Umeh & Odibo (2014) reported that complete retting of fresh cassava roots results in high yield of wet *fufu* mash and enhances detoxification. The study by Otoo *et* *al*. (2018) revealed that the reduced pH achieved through retting impacts sour taste and characteristic aroma to *fufu*. However, prolonged retting and reduced starch content of the fresh cassava roots have been reported to result in intense *fufu* odour that is usually undesirable to consumers (Achi & Akomas, 2006; Bechoff *et* *al*., 2018). Retting has also been linked to textural properties of the cooked *fufu*. Isirima *et* *al*. (2018) observed a higher and better index for drawability, mouldability, smoothness and colour in *fufu* processed using the retting method compared with other cassava processing methods. The improved textural property of *fufu* processed using retting method is linked to the breaking up of carbohydrate granules to smaller particles through the disintegration of building molecules in the cassava (Isirima *et* *al*., 2018). The author further revealed that incomplete break down or absence of retting resulted in products with coarse granules or particles. Hongbete *et* *al*. (2009) and Bindzi *et* *al*. (2014) furthermore reported that leaching out of phenol during soaking (retting) and dewatering of cassava roots enhance the white colour of cassava products such as flour and cooked *fufu*. This study therefore reveals that the trait ‘high retting ability’ influences most of the organoleptic properties of *fufu* such as colour, aroma, texture and overall acceptance of the end product, and hence its importance to the respondents.

### High and low processing properties of fermented fufu mash for cooking of fufu

**Figure 5:**
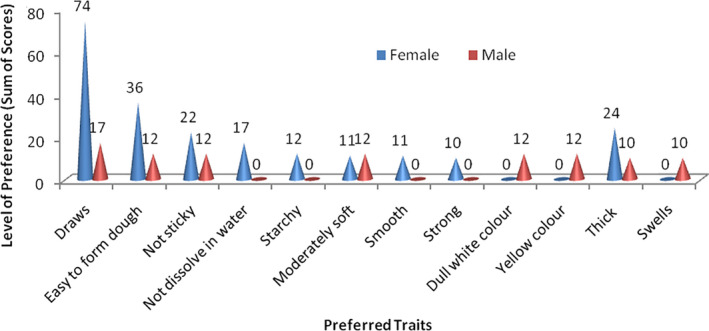
Preferred characteristics during fufu preparation.

With regard to the preferred cooking properties of the intermediate *fufu* product (fermented wet *fufu* mash), Fig. 5 shows that the trait ‘easy to form dough’ is of key interest (high sum of scores) to both the male (12) and female respondents (36); however, ‘drawing ability’ and ‘thickness’ of the wet mash during cooking were prioritised by the women (74) and (24) respectively, who also are the key actors at this level of *fufu* processing. According to the respondents in our survey, ‘dough formation’ of mash is related to health status of the crop indicated by the absence of pest and diseases. This is in agreement with previous study by Numfor (1999), who had also related dough formation ability of *fufu* mash to health of the cassava variety used during processing. This highly prioritised trait ‘easy to form dough’ may also be related to ease of gelatinisation and pasting properties of the fermented *fufu* mash. According to Bechoff *et* *al*. (2017), fast gelatinisation of the starch paste is the trait that is highly appreciated by *lafun* and *fufu* processors. Gelatinisation that is manifested by swelling, disruption of hydrogen bonds, crystallite melting with subsequent disappearance of Maltese cross, viscosity development, and starch molecules solubilisation is accompanied by changes in viscosity and formation of paste. On the other hand, pasting properties of *fufu* flour are important quality indices in predicting the behaviour of *fufu* paste during and after cooking (Etudaiye *et* *al*., 2008). The study by Bindzi *et* *al*. (2014) reported that pasting temperatures are indicative of the minimum energy required to initiate rapid absorption of water and swelling of starch granules resulting in increased viscosity and formation of dough. Pasting temperature gives an indication of the minimum temperature for cooking of a given sample (Bindzi*et* *al*., 2014). The study by Chisenga *et* *al*. (2019) further revealed that pasting property of starch, defined by the pasting temperatures and viscosities, is affected by amylose content and proportion of components in the food matrix. This study therefore suggests that the trait ‘easy formation of dough’ is related to lower pasting temperature and time, and enhanced by the utilisation of cassava varieties that are free of pest and diseases. Cassava varieties with such quality traits will therefore require reduced processing time and labour, and hence its importance to the respondents.

The study further reveals that the trait ‘thickness’ of *fufu* mash during processing is another determinant of preference especially among the female respondents. This may be referred to the gel strength and viscosity of the aqueous fermented starch paste, which develops during stirring and cooking of final product. This trait can be characterised using pasting and rheological or textural properties of the thick cassava slurry obtained from the fermented *fufu* mash. This female‐preferred processing trait may be influenced negatively by the other female‐preferred trait (high retting ability). According to Nkoudou *et* *al*. (2020), accelerated retting process reduces the thickening power of the derived cassava flour. The author further reported that degradation rate and the softening degree of cassava roots influences viscosity; accelerated retting process results in reduced viscosity. Thickness of *fufu* mash may also facilitate formation of dough of the mash and reduce cooking time.

The preferred processing trait, ‘drawing ability’ may be likened to the degree of cohesiveness of the *fufu* paste during cooking. According to Bechoff *et* *al*. (2018), cohesiveness, a major textural property that drives consumer acceptance, is linked to starch composition especially amylose content. The earlier findings by Numfor (1999) and Rosales‐soto *et* *al*. (2016) indicated that cohesiveness in cassava paste is associated with intermolecular forces within the food, and failure of starch granules to release sufficient amylose. This leads to reduction in cohesiveness of fermented cassava product. In addition, Dufour *et* *al*. (2002) reported that presence of fibre can reduce the cohesiveness of cassava paste. These textural and pasting characteristics of starch have been associated with cooking quality and texture of various food products (Otegbayo *et* *al*., 2006). The preferred trait (drawing ability) may have a bearing on retting ability of the cassava roots. Isirima *et* *al*. (2018) reported an acceptable degree of stickiness, mouldability and drawability for *fufu* made from flour processed by retting compared with those from blanching and direct method of cassava processing.

### Preferred and less‐preferred quality characteristics of fufu during consumption

**Figure 6:**
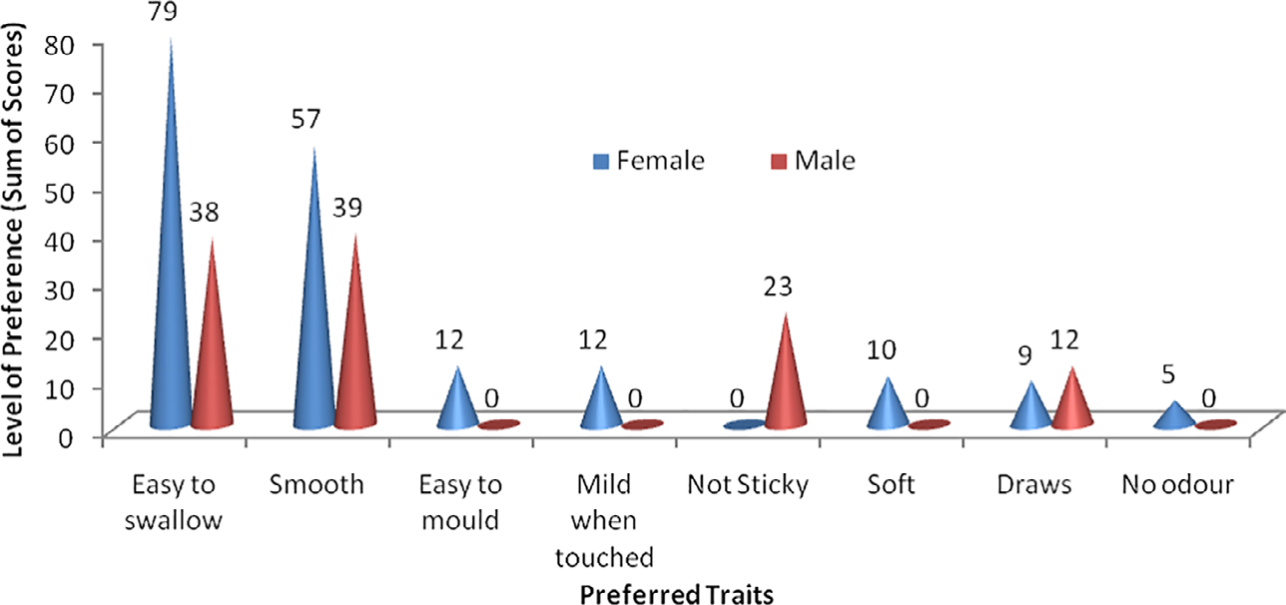
Preferred characteristics of fufu during consumption.

Two quality traits, ‘*fufu* smoothness’ and ‘easy to swallow’ were of utmost importance (highest sum of scores) to male and female *fufu* consumers in South‐East Nigeria (Fig. 6). Our study however shows that female respondents (57) placed higher emphasis on *fufu* smoothness compared with their male counterparts (39). Similarly, the trait ‘easy to swallow’ was assigned higher weight by greater number of the females (79) than males (38). Bechoff *et* *al*. (2018) described smooth *fufu* as dough that is homogeneous in appearance and hand‐feel, and does not have notable fibres, lumps or particles. The author further reveals that *fufu* smoothness is enhanced by the removal of fibre during the sieving operation of fermented roots or mash. This further corroborates the report by Uyoh *et* *al*. (2009) who stated that insoluble fibres are disintegrated by cellulolytic and pectinolytic enzymes activities during retting, hence enhancing efficiency of sieving and smoothness of *fufu*. This finding suggests that smoothness of *fufu* may be correlated with the fibre content of the fresh cassava root. Our study further revealed that smoothness of *fufu* was also associated with the trait ‘easy to swallow’ by the male respondents, implying that the two terms are related. Dziedzoave *et* *al*. (1999) also related smoothness to uniformity of particles. Hence, the highly preferred textural attributes of cooked *fufu* ‘smoothness’ and ‘easy to swallow’ could be related to biophysical properties of the fresh cassava root such as fibre content and efficiency of the unit operation ‘retting’ and ‘sieving’ during *fufu* processing.

**Figure 7:**
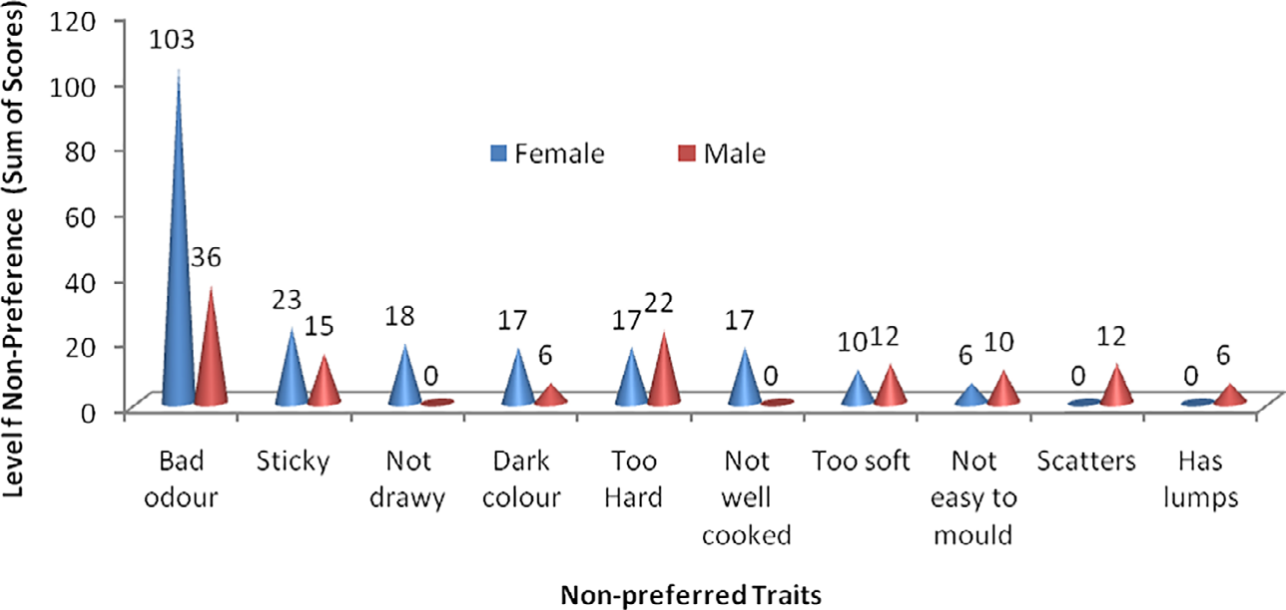
Less‐preferred characteristics of fufu for consumption.

In contrast, the respondents associated two outstanding traits (stickiness and offensive odour) with low‐quality attributes of cooked *fufu* (Fig. 7). Stickiness in cooked *fufu* seems to be a trait of key importance to female consumers (23) compared with the male respondents (15). Stickiness could be likened to the textural attribute ‘adhesiveness’, which is described as the work required in overcoming the attractive force between a product and the contact surface (Singh *et* *al*., 2013). Bechoff *et* *al*. (2018) reported high elastic and sticky texture in *eba* and *fufu* prepared from white flesh cassava varieties compared with yellow flesh varieties. This was attributed to their high dry matter and starch contents. The study also shows that ‘intense *fufu* odour’ is a less‐preferred trait mainly for female *fufu* consumers (103). However, fewer male respondents (33) indicated that odour of *fufu* lowers their preference for the product. This is in agreement with the report by Uyoh *et* *al*. (2009) who stated that one major problem in processed *fufu* is the flavour of the product, which may be unacceptable to many people. Shittu & Adedokun (2010) and Tomlins *et* *al*. (2007) further indicated that the acceptability of *fufu* by consumers is related to its characteristic aroma. Increased fermentation time had already been reported to correlate positively with the intensity of *fufu* aroma (Achi & Akomas, 2006; Bechoff *et* *al*., 2018). However, Bechoff *et* *al*. (2018) reported a negative correlation between starch content of different cassava varieties and intensity of typical *fufu* aroma.

This study therefore shows that ‘smoothness’, ‘easy to swallow’, ‘bad odour’ and ‘stickiness’ are the main traits that positively or negatively influence consumption of cooked *fufu* in South‐East Nigeria. The quality attributes ‘stickiness’ and ‘easy to swallow’ are related to the sensory property and texture, while the traits ‘bad odour’ and ‘smoothness’ are related to aroma and appearance, respectively. The study also shows that these sensory attributes of *fufu* may be related to the processing parameters ‘retting’ and ‘sieving’.

## Conclusion

The study analysed consumer preferences and quality attributes of *fufu* in the South‐East region of Nigeria between men and women. Major preferred root quality traits identified in locally preferred varieties [*Dabere*, *Imo* *best*, *Torokwem*, *Gbayuomma*, *Aguoegbulam*, *akwatakwa*, *Akpalam* *Aka*, *Codelia*, *Ogwuru* *ego*, *Mmaduabuchi*, *Sakasaka*, *Akpu* *da* *grace*, *Nwaocha*, *Nwaibibi*, *Nwanyiumuahia* (lady from Umuahia), Yellow root (Vit. C)], TMEB 419, Agric., and TMS 98/0505, within the study area were as follows: root size, heaviness, appearance/smoothness of outer root skin and inner root flesh colour. ‘Ease of peeling’, ‘freshness of root’ and ‘high retting ability’ (easy to ferment) were identified as the preferred processing traits for the fermented *fufu* mash, while ‘easy to form dough’, ‘thickness’ and ‘drawing ability’ stood out as the traits of preference during cooking of the fermented wet *fufu* mash. At the point of consumption, ‘smoothness’ and ‘easy to swallow’ were identified as the major determinants of preference, while ‘stickiness’ and ‘intense *fufu* aroma’ were identified as less‐preferred traits. The study therefore reveals that the preference of these traits by the female respondents was directly linked to the processing parameters, and overall quality of the end product. On the contrary, economic gain was observed to be the key determinant driving the choice of the male respondents. The study further indicated that the preferred root qualities ‘root size, heaviness’, ‘appearance/smoothness of outer root skin’ and ‘inner root flesh colour have effect on the processing trait ‘ease of peeling’, and on the colour and yield of the fermented *fufu* mash, and cooked dough. The study further suggests that the preferred processing traits ‘ease of peeling’, ‘freshness of root’ and ‘high retting ability’ (easy to ferment) are intertwined and are associated with colour, yield, organoleptic properties and overall acceptance of cooked *fufu*. However, the retting ability of cassava roots was observed to be a major factor influencing most of the processing traits and those of the cooked product. Our finding therefore reveals an undeniable link between the preferred root quality traits, preferred processing traits and overall quality of cooked *fufu*. Emphasis on root size, heaviness, appearance and root flesh colour, and the relation of these traits to preferred processing and *fufu* quality attributes provides insight for cassava breeders on areas to redirect breeding activities. These findings therefore make it imperative for introduction of a multidisciplinary, multistakeholder approach into crop improvement programmes, especially the science behind food processing. This will assist in breeding of varieties that meet end user needs and enhance adoption of these new varieties. The female‐preferred traits should be of utmost importance to the breeder, since they are the key actors in the processing operations. There may be need to conduct scientific investigation using throughput methods such as NIRS to identify the biochemical properties that affect these quality traits.

## Conflict of interest

There is no conflict of interest among authors of this paper.

## Author contribution

**Ugo Chijioke:** Conceptualization (equal); Data curation (equal); Investigation (equal); Project administration (equal); Supervision (equal); Validation (equal); Visualization (equal); Writing‐original draft (equal); Writing‐review & editing (equal). **Tessy Madu:** Data curation (equal); Investigation (equal); Methodology (equal); Supervision (equal); Writing‐original draft (equal); Writing‐review & editing (equal). **Benjamin Okoye:** Conceptualization (equal); Data curation (equal); Formal analysis (equal); Software (equal); Validation (equal); Writing‐original draft (equal); Writing‐review & editing (equal). **Amaka Promise Ogunka:** Investigation (equal); Validation (equal); Writing‐original draft (equal); Writing‐review & editing (equal). **Mercy Ejechi:** Investigation (equal); Methodology (equal); Validation (equal); Visualization (equal); Writing‐original draft (equal); Writing‐review & editing (equal). **Miriam Ofoeze:** Investigation (equal); Writing‐original draft (equal); Writing‐review & editing (equal). **Chukwudi Ogbete:** Investigation (equal); Writing‐original draft (equal). **Damian Njoku:** Validation (equal); Writing‐original draft (equal); Writing‐review & editing (equal). **Justin Ewuziem:** Investigation (equal); Methodology (equal); Writing‐review & editing (equal). **Confidence Kalu:** Investigation (equal); Methodology (equal); Writing‐review & editing (equal). **Nnaemeka Onyemauwa:** Formal analysis (equal); Investigation (equal); Writing‐review & editing (equal). **Blessing Ukeje:** Data curation (equal); Writing‐review & editing (equal). **Oluchi Achonwa:** Validation (equal); Writing‐original draft (equal); Writing‐review & editing (equal). **Lora Forsythe:** Data curation (equal); Methodology (equal). **Genevieve Fliedel:** Data curation (equal); Formal analysis (equal); Methodology (equal); Writing‐review & editing (equal). **Chiedozi Egesi:** Supervision (equal); Validation (equal); Writing‐review & editing (equal).

## Ethical approval

This study was assessed and approved by the National Research Ethics Committee. Research teams obtained ethical approval prior to the fieldwork. Participants were informed about the study and explained that their participation was entirely voluntary, that they could stop the interview at any point and that the responses would be anonymous. Written consent (signature) was sought and obtained from respondents participating in this study.

### Peer review

The peer review history for this article is available at https://publons.com/publon/10.1111/ijfs.14875.

## Supporting information


**Table S1**. Gender Profiling of Raw material characteristics: for product quality (agronomic, post‐harvest).

## Data Availability

Research data are not shared
