# Supplementary material for: Quality attributes of fufu in South‐East Nigeria: guide for cassava breeders
Source: Int J Food Sci Technol. 2020 Dec 16;56(3):1247–57. doi: 10.1111/ijfs.14875 (PMC7986842; doi:10.1111/ijfs.14875)
Supplement: Supplementary file 1 — Table S1. Gender Profiling of Raw material characteristics: for product quality (agronomic, post‐harvest). [file IJFS-56-1247-s001.doc]

**Supplementary Table 1: Gender Profiling of Raw material characteristics: for product quality (agronomic, post-harvest)**

| **Gender profiling** | |
| --- | --- |
| - Are there any gender differences in characteristic preferences or rankings that are potentially problematic? Any trade-offs or conflicts? E.g. high starch content in cassava may be a bad or good for different products, small/large roots, hard or soft. | Big/large Roots: The women find it difficult to peel because it takes their time and also during soaking for fufu production, they have to cut it into pieces. Also some said big roots are full of water, has high moisture and affects the fufu yield.  Small roots: The women also said the small roots are difficult to peel which causes peel loss.  Both Men and Women complained about low starch content in Cassava especially the yellow root Which affects the level of demand for such fufu and also, the yellow colour makes the fufu looks like gari and about 90% Fufu consumers prefer the white coloured fufu.  Poor adoption rate: inability of the variety to adapt to their soil might cause low adoption rate. The improved varieties not able to meet up with the expected product quality which will affect the demand and supply of the product. |
| **Type of evidence** |  |
| - What (+/-) influence does the characteristic potentially have on women’s workload drudgery, time, or level exertion, and why?   Refer to EVIDENCE, link to the questions in the SOK and the activity 3 report. | **Big Roots**- it will make for ease of peeling. It might affect the fermentation time/period.  **Many roots**- It will take time in peeling.  It will yield more and cost more time to peel.  **Strong root/difficult to penetrate fingernails**: It will take a longer period to ferment and all the roots might not ferment which will lead to loss of material, valuable time and money.. It gives them more yield.  **Easy to peel**: it will reduce time and drudgery.  Freshly harvested root: Easy to peel and high moisture depending on the variety.  -**Not well dewatered mash** will affect the texture of the fufu either to make it too soft. |
| **Prioritized Traits of Importance** | |
| - Identify the key indicators that accompany the main characteristics mentioned | - The roots are usually big (Big like or even bigger than eva bottled water (1Ltr) that when you harvest 4-5 stands your basin will be filled. - When you look at the tubers, you see fresh milky fluid at the head (milk colour). - The root must be fresh to enable fermentation. - The skin will be fresh. - The cassava tubers should be freshly harvested. - the skin of the root should be hard to touch and not watery - The peeling should be easy while peeling - good colour with no extra colour (either white or yellow), white mostly preferred. - The Cassava should ferment within 3 days. - It will have a creamy colour without dirt particles - the inner membrane should not be foamy (like bread) - Good quantity of mash after grating and after sieving. - Colour should be milky and not white like plain sheet - The fufu should be neat without particles. - The fufu will draw to show it was well cooked. - When touch, it will not stick to your hand |
| - Identify the good varieties that accompany the main high characteristics (including market interviews) | **Good Varieties:**  TME 419 (N**I)**, Dabere(L), TMS98/0505 (I), Imo best(I), Torokwem(L), Gbayuomma (L) , Aguoegbulam(L), yellow root(I), akwatakwa(L), agric.(I),Akpalam Aka, Codelia, Ogwuru ego, Mmaduabuchi, Sakasaka, Akpu da grace, Nwaocha, Nwaibibi, nwanyiumuahia (lady from Umuahia)(I), Yellow root (Vit. C) NR |
